# Antioxidant Effect of Curcumin and Its Impact on Mitochondria: Evidence from Biological Models

**DOI:** 10.3390/jox15050139

**Published:** 2025-08-31

**Authors:** Karla Alejandra Avendaño-Briseño, Jorge Escutia-Martínez, Estefani Yaquelin Hernández-Cruz, José Pedraza-Chaverri

**Affiliations:** 1 Laboratory F-315, Department of Biology, Faculty of Chemistry, National Autonomous University of Mexico, Mexico City 04510, Mexico; marsave52@gmail.com (K.A.A.-B.); escutiajorge@gmail.com (J.E.-M.); 2 Graduate Program in Biological Sciences, National Autonomous University of Mexico, University City, Mexico City 04510, Mexico

**Keywords:** curcumin, mitochondria, reactive oxygen species, antioxidants

## Abstract

Curcumin, the principal active component of turmeric, is a polyphenol that has been used in various countries for the treatment of numerous conditions due to its wide range of health benefits. Curcumin exhibits bifunctional antioxidant properties: the first is attributed to its chemical structure, which enables it to directly neutralize reactive oxygen species (ROS); the second is related to its ability to induce the expression of antioxidant enzymes via the transcription factor nuclear factor erythroid 2–related factor 2 (Nrf2). Both ROS and Nrf2 are closely associated with mitochondrial function and metabolism, and their dysregulation may lead to mitochondrial dysfunction, potentially contributing to the development of various pathological conditions. Therefore, curcumin treatment appears highly promising and is strongly associated with the preservation of mitochondrial function. The aim of this review is to summarize the current literature on the impact of curcumin’s antioxidant properties on mitochondrial function. Specifically, studies conducted in different biological models are included, with emphasis on aspects such as mitochondrial respiration, antioxidant enzyme activity, interactions with mitochondrial membranes, and the role of curcumin in the regulation of intrinsic apoptosis.

## 1. Introduction

The turmeric (*Curcuma longa*), also known as Indian saffron, is a plant that has been used for many years in traditional Asian medicine, in addition to its use as a spice and coloring agent. This plant belongs to the same family as ginger, is native to India and Southeast Asia, and is currently cultivated in tropical and subtropical regions [1].

Historically, the use of the plant has been limited primarily to the rhizomes, with the leaves often discarded during processing. However, recent studies have demonstrated that the leaves of *Curcuma longa* are a valuable source of protein, fiber, and bioactive compounds, and contain significant concentrations of trace elements such as sodium (Na), magnesium (Mg), calcium (Ca), and manganese (Mn) [2].

The underground stem of the plant consists mainly of water (approximately 80%), followed by carbohydrates (13%), proteins (2%), minerals (2%), lipids (1%), and curcuminoids (3–5%). Curcuminoids are a group of phenolic phytochemicals, including curcumin, that share a diarylheptanoid structure, comprising two aromatic rings connected by a seven-carbon linker, with various potential substituents [3]. These compounds are known for a broad spectrum of biological activities, including anti-inflammatory [4], antioxidant [5], antimicrobial [6], and antitumor effects [7].

The three major curcuminoids are curcumin (approximately 70%), demethoxycurcumin (17%), and bisdemethoxycurcumin (3–6%) [8]. Although curcuminoids are found in other species of the Zingiberaceae family, such as *Curcuma zedoaria* and *Curcuma aromatica*, *Curcuma longa* remains the most prominent and studied source [9].

Due to its high natural abundance, curcumin is more easily isolated compared to other curcuminoids, which has facilitated its widespread use in experimental studies. These investigations have revealed a wide range of biological effects beyond those already mentioned, including immunomodulatory [10], hepatoprotective [11], and neuroprotective [12] activities. Curcumin has been shown to act on various cell types and, in some cases, its molecular targets have been identified, including growth factors, transcription factors, redox-regulating enzymes, and cell surface receptors.

Curcumin is recognized and used in multiple countries in diverse forms due to its extensive range of health benefits. A clinical trial has demonstrated that curcumin is well-tolerated and safe in pancreatic cancer patients, even at doses up to 8000 mg/day [13]. However, to date, the United States Food and Drug Administration (FDA) has not approved curcumin as a therapeutic agent for any condition; it remains classified as a safe food additive.

This review aims to provide a comprehensive overview of curcumin, focusing on its chemical structure, metabolism, and antioxidant mechanisms of action. Furthermore, it summarizes the current literature on the impact of curcumin’s antioxidant properties on mitochondrial function, highlighting findings from studies conducted in various biological models. Particular attention is given to aspects such as mitochondrial respiration, antioxidant enzyme activity, interactions with mitochondrial membranes, and curcumin’s role in regulating intrinsic apoptotic pathways.

## 2. Curcumin

**Figure 1:**
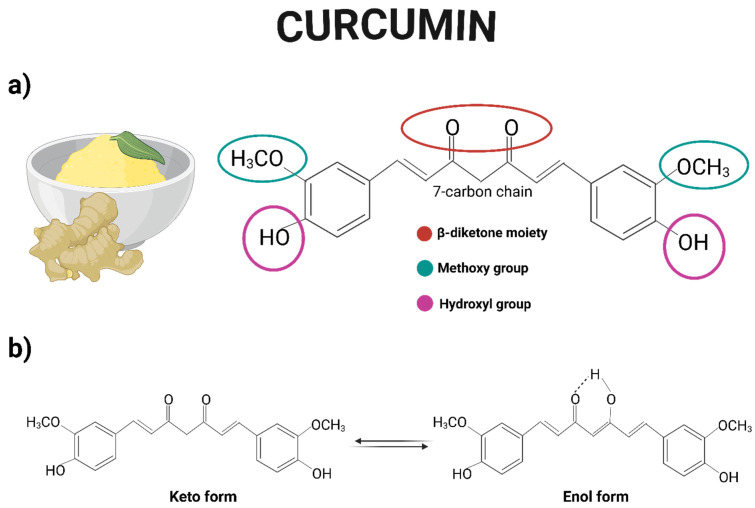
(**a**) Chemical structure of curcumin, highlighting the hydroxyl and methoxy functional groups, as well as the β-diketone moiety. (**b**) Representation of the keto-enol tautomerism of curcumin. Created with BioRender.com.

Curcumin is a yellow-orange solid compound widely used as a food additive and coloring agent. Also known as diferuloylmethane, curcumin is a symmetrical molecule belonging to the diarylheptanoid group. Chemically, it is a polyphenol composed of two aromatic rings, each bearing a hydroxyl and a methoxy group as substituents. These rings are connected by a seven-carbon chain that includes two carbonyl groups located at positions 3 and 5 (commonly referred to as the β-diketone moiety), which can undergo keto-enol tautomerism [14] (Figure 1). Its chemical formula is C_21_H_20_O_6_, with a molecular weight of 368 g/moL and a melting point of 183 °C [15]. Curcumin is practically insoluble in water but is readily soluble in organic solvents such as DMSO, methanol, ethanol, and chloroform [16].

Curcumin is a photosensitive molecule, and exposure to light is the main route of its degradation. Although the photodegradation mechanism is not yet fully understood, it has been proposed that it primarily involves the cleavage of the β-diketone moiety, leading to the formation of smaller phenolic compounds. Among the colorless degradation products identified in this process are vanillin and ferulic acid [17].

The liver is considered the primary site of curcumin metabolism, along with the intestine. Curcumin undergoes both phase I and phase II biotransformation. Phase I metabolism involves the action of reductases, which reduce the double bonds in the molecule, converting curcumin into dihydrocurcumin, tetrahydrocurcumin, hexahydrocurcumin, and octahydrocurcumin. In phase II metabolism, both curcumin and its reduced metabolites can be conjugated with glucuronic acid and sulfate at the phenolic site [18,19]. Additionally, curcumin may be sulfated in the cytosol, primarily by phenol sulfotransferase isoenzymes SULT1A1 and SULT1A3 [20]; meanwhile, uridine 5’-diphospho-glucuronosyltransferases (UGTs), mainly UGT1A1, UGT1A8, and UGT1A10, catalyze glucuronidation in intestinal and hepatic microsomes [21].

Moreover, it has been proposed that curcumin can be alternatively metabolized by the gut microbiota. In 2011, Hassanniaasab and colleagues [22] screened human fecal microorganisms for curcumin-converting activity and successfully isolated *Escherichia coli*, which exhibited the highest activity among the Enterobacteriaceae tested. The responsible enzyme was later isolated, purified, and characterized from *E. coli* strain K-12, which showed the highest curcumin-converting capacity. The researchers identified the enzyme as an NADPH-dependent reductase that catalyzes a two-step reduction pathway, beginning with the conversion of curcumin to dihydrocurcumin and subsequently to tetrahydrocurcumin as the final product [22].

## 3. Strategies to Enhance the Bioavailability of Curcumin

Despite its beneficial effects, the most frequent criticism of curcumin consumption is its poor systemic bioavailability. Numerous studies have reported low or even undetectable blood concentrations following oral administration of curcumin. The main reasons for this appear to be the chemical instability of the molecule, poor absorption, and rapid systemic elimination.

To address these limitations, various strategies have been developed to enhance curcumin’s bioavailability. One of the most well-known approaches involves co-administration with compounds that inhibit or delay curcumin metabolism. Piperine, the major active component of black pepper, is a well-established inhibitor of hepatic and intestinal glucuronidation. A pioneering study conducted in 1998 by Shoba et al. [23] demonstrated that co-administration of piperine (20 mg/kg) with curcumin (2 mg/kg) significantly enhanced the bioavailability of the polyphenol. In animal models (rats), bioavailability increased by 154%, while in humans, it rose by as much as 2000%, with no reported adverse effects associated with the combination [23]. Twelve years later, Suresh and Srinivasan [24] confirmed these findings in rats, showing significantly improved bioavailability of curcumin when administered orally alongside piperine. This enhancement was attributed to improved intestinal absorption and prolonged tissue retention of curcumin, with intact curcumin detected in the brain up to 96 h after administration.

Other strategies to improve curcumin bioavailability include the use of nanoparticles. Various formulations, such as those based on high molecular weight poly (lactic-co-glycolic acid) (PLGA) and coatings with saponins, have shown enhanced absorption and stability of curcumin in animal models [25,26].

In addition to the aforementioned approaches, alternative routes of administration have been explored, including subcutaneous, intraperitoneal, and intravenous injection. In general, these methods have yielded promising results for increasing the bioavailability of curcumin, thereby enhancing its therapeutic effects across a wide range of diseases. For instance, a single subcutaneous dose of PLGA microparticles in mice was able to maintain detectable curcumin levels in blood and various tissues for nearly a month. Furthermore, this formulation exhibited strong anticancer activity in immunodeficient mice bearing MDA-MB-231 human breast cancer xenografts and significantly reduced tumor angiogenesis [27]. Likewise, intravenous injection of curcumin formulated in human serum albumin nanoparticles enhanced the anticancer efficacy of curcumin in xenografts of human HT116 colon cancer cells [28].

Interestingly, a marked difference in curcumin’s effectiveness was observed in the same study depending on the route of administration used to attenuate bleomycin-induced lung injury. Intraperitoneal administration was significantly more effective than oral delivery. The authors hypothesized that curcumin bioavailability varied significantly with the route of administration. To test this hypothesis, they measured plasma curcumin levels in mice using high-performance liquid chromatography (HPLC) following oral or intraperitoneal administration of curcumin (300 mg/kg body weight). Plasma collected two hours after oral dosing showed a concentration of 15.7 ± 3.8 ng/mL (approximately 43 nM), whereas intraperitoneal administration resulted in a much higher plasma concentration of 181 ± 23.1 ng/mL (506 nM). Therefore, intraperitoneal delivery of curcumin led to higher plasma concentrations and a greater effective systemic dose than oral administration [29].

Despite these advances, intraperitoneal, intravenous, and subcutaneous delivery of curcumin have been more commonly studied in animals. Further research is needed to validate their efficacy and safety in humans. Oral administration remains the preferred option due to its convenience, high compliance, and strong patient acceptance.

## 4. Antioxidant Activity of Curcumin

Curcumin is a bifunctional antioxidant, as it has the ability to directly react with reactive oxygen species (ROS), and additionally, it can induce the expression of cytoprotective and antioxidant proteins through the nuclear factor erythroid 2–related factor 2 (Nrf2) pathway.

The chemical structure of curcumin determines its antioxidant activity, as the phenolic hydroxyl (OH) groups are involved in the scavenging or neutralization of free radicals [30,31]. These OH groups donate a hydrogen atom to the radicals, thereby reducing their reactivity. As a result, a relatively stable oxidized product known as the phenoxyl ion/radical is formed [32].

Moreover, the β-diketone moiety of curcumin enables it to react with metals to form complexes. These complexes not only alter the physicochemical properties of curcumin but also affect the biological reactivity of the metals involved [33,34]. Several studies have demonstrated that curcumin-metal complexes possess greater antioxidant capacity than curcumin alone [35,36,37]. For instance, in a study conducted by Thakam and Saewan [35], curcumin was complexed with bivalent metal ions (Zn^2+^, Se^2+^, Cu^2+^, Fe^2+^, Mg^2+^, and Mn^2+^), and the resulting complexes were characterized and evaluated for their radical-scavenging activity. The researchers reported that the complexes showed superior antioxidant performance compared to curcumin alone, with zinc being the most effective in enhancing antioxidant capacity.

**Figure 2:**
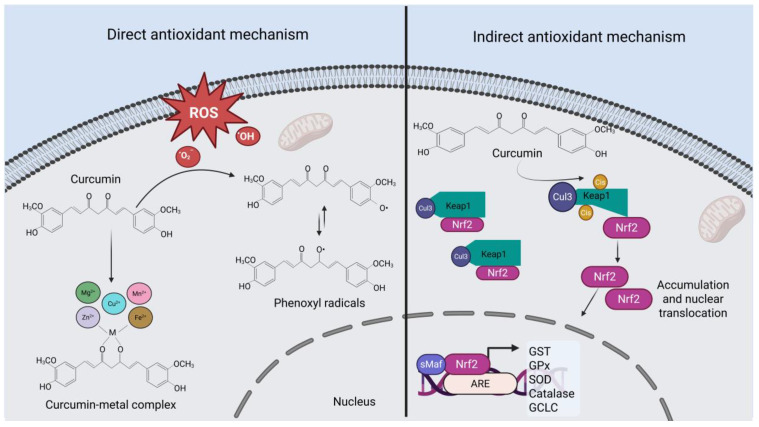
Antioxidant mechanism of curcumin. The direct mechanism (left panel) relies on the phenolic OH groups of curcumin, which act as electron donors to neutralize free radicals. This leads to the formation of phenoxyl radicals, which are relatively stable. The β-diketone moiety enables curcumin to interact with metals, thereby modifying their biological reactivity. On the other hand, the indirect antioxidant mechanism (right panel) is mediated by the activation of the transcription factor Nrf2, promoting the expression of endogenous antioxidant enzymes. ARE, Antioxidant Response Elements; Cis, Cysteines; Cu^2+^, Copper; CUL3, Cullin E3 Ubiquitin Ligase; ROS, Reactive Oxygen Species; Fe^2+^, Iron; GCLC, Glutamate–Cysteine Ligase; GST, Glutathione S-Transferase; GPX, Glutathione Peroxidase; H_2_O_2_, Hydrogen Peroxide; Keap1, Kelch-like ECH-associated Protein 1; Mg^2+^, Magnesium; Mn^2+^, Manganese; Nrf2, Nuclear Factor Erythroid 2–Related Factor 2; O_2_^−^, Superoxide Radical; SOD, Superoxide Dismutase. Created with BioRender.com.

On the other hand, Nrf2 is the master transcriptional regulator of cellular responses to oxidative stress. It is well established that various chemical and natural inducers (including curcumin) enhance endogenous antioxidant defenses through the activation of Nrf2. Multiple cellular factors are involved in the regulation of the stability and activation (i.e., nuclear translocation) of this protein [38]. The most well-known regulatory mechanism is described below:

Under normal homeostatic conditions, the Kelch-like ECH-associated protein 1 (Keap1) anchors Nrf2 in the cytosol and facilitates its degradation. Keap1 acts as an adaptor for the Cullin 3 (Cul3)-based E3 ubiquitin ligase complex, promoting Nrf2 ubiquitination and subsequent proteasomal degradation, thereby preventing its activation. However, curcumin induces the modification of cysteine residue 151 in Keap1, leading to conformational changes that hinder Keap1’s ability to mediate Nrf2 ubiquitination by the Cul3 complex. As a result, Nrf2 accumulates, stabilizes, and translocates into the nucleus, where it dimerizes with small proteins (such as Maf or Jun). This dimerization allows Nrf2 to bind to antioxidant response elements (AREs), activating the transcription of a variety of genes encoding cytoprotective, antioxidant, and phase II detoxification proteins [39,40,41,42]. Figure 2 summarizes the antioxidant mechanisms of action of curcumin, including both direct and indirect pathways.

## 5. Curcumin and the Mitochondrion

Mitochondria play a central role in cellular metabolism by generating the majority of adenosine triphosphate (ATP) through the catabolism of nutrients. This is accomplished via mitochondrial respiration, a complex process that includes the tricarboxylic acid (TCA) cycle (also known as the Krebs cycle). The TCA cycle takes place in the mitochondrial matrix and its primary functions include the production of guanosine triphosphate (GTP), which is energetically equivalent to ATP, as well as the generation of the electron carrier’s nicotinamide adenine dinucleotide in its reduced form (NADH) and flavin adenine dinucleotide in its reduced form (FADH_2_). These carriers shuttle electrons to the electron-transport chain, a series of protein complexes embedded in the inner mitochondrial membrane. As electrons traverse the chain, protons are pumped into the intermembrane space, creating an electrochemical gradient that ultimately drives ATP synthesis [43].

Given its pivotal role in energy production, mitochondrial integrity is essential to cellular homeostasis. The following section examines the involvement of mitochondria in the etiology of various diseases, highlighting the impact of mitochondrial genome mutations, the generation of and defense against ROS, and mitochondrial contributions to biosynthetic and signaling pathways, as well as the modulatory effects of curcumin on these functions.

### 5.1. Role of Mitochondria in the Etiology of Diseases

ATP synthesis is an intricate process; even minor disruptions can precipitate a variety of diseases, particularly in high-energy-demand tissues such as brain and skeletal muscle. Some mitochondrial dysfunctions arise from inherited or acquired mutations in the mitochondrial genome. Such mutations may disrupt overall mitochondrial protein synthesis, via large-scale deletions of mitochondrial DNA or point mutations in specific genes. Notably, pathogenic mutations in mitochondrial tRNA and rRNA genes often impair the translation of the thirteen polypeptides encoded by mitochondrial DNA, leading to defects in oxidative phosphorylation. For example, mutations in the mitochondrial tRNA^Lys gene are frequently associated with epilepsy, elongated muscle fibers, and myoclonus (involuntary, jerky muscle contractions) [44].

Oxygen serves as the terminal electron acceptor in the respiratory chain, combining with protons and electrons to form water according to the reaction:

O_2_ + 4H^+^ + 4e^−^ ⟶ 2H_2_O

However, mitochondria are also a major source of intracellular ROS, generated when electrons “leak” from the transport chain and partially reduce oxygen. Under physiological conditions, an estimated 0.2–2% of electrons escape the normal transfer pathway, directly reacting with oxygen to produce superoxide (O_2_^−^), a highly reactive species [45]. The O_2_^−^ is subsequently converted into hydrogen peroxide (H_2_O_2_) and other ROS. At low concentrations, ROS function as signaling molecules that regulate processes such as cell differentiation; but uncontrolled ROS production can inflict both mitochondrial and widespread cellular damage [46].

To mitigate ROS, mitochondria possess antioxidant defense systems, most prominently superoxide dismutase (SOD), catalase (CAT), and glutathione peroxidase (GPx), which neutralize ROS before they cause harm [47]. When these defenses are overwhelmed, excessive ROS provoke oxidative damage to mitochondrial proteins, DNA, and lipids, impairing respiratory-chain enzyme activities, interrupting ATP production, and compromising other essential functions. Such mitochondrial dysfunction contributes to a range of pathological conditions, including aging and metabolic diseases [48].

Beyond energy generation, mitochondria supply key precursors for macromolecular biosynthesis, participating in the synthesis of nucleotides, fatty acids, cholesterol, amino acids, and heme. A central example is citrate, a TCA-cycle intermediate that not only regulates energy production but also modulates anabolic pathways. Following its synthesis from acetyl-CoA and oxaloacetate by citrate synthase, citrate may either continue through the TCA cycle or be exported to the cytosol via a specific transporter, depending on the cell’s energetic and biosynthetic state. High cytosolic citrate levels inhibit glycolysis, via allosteric suppression of phosphofructokinase-1, and stimulate ATP-consuming pathways such as lipid synthesis. In the cytosol, ATP-citrate lyase cleaves citrate into oxaloacetate and acetyl-CoA, the latter being the key precursor for fatty-acid and sterol biosynthesis. Moreover, certain metabolites and their concentrations act as signals to reshape gene expression; in this context, newly generated acetyl-CoA plays a significant role in epigenetic modifications, such as histone acetylation [49,50]. These biosynthetic pathways are integral to stress responses and are often dysregulated in disease.

Finally, mitochondria serve as environmental sensors that communicate with other subcellular compartments. A principal mode of communication is through physical contacts at mitochondrial-associated membranes (MAMs), specialized contact sites between mitochondria and the endoplasmic reticulum. MAMs form a crucial platform for interorganelle molecule exchange and cross-talk, governing calcium homeostasis, lipid metabolism, and apoptosis regulation. Disruption of MAM composition or aberrant MAM formation is linked to various pathologies, notably neurological and cardiovascular diseases [51,52,53]

Together, these pleiotropic mitochondrial functions underscore how subtle perturbations in organelle structure or activity can disrupt cellular homeostasis and contribute to the pathogenesis of numerous diseases.

### 5.2. Effects of Curcumin on Mitochondria

#### 5.2.1. Mitochondrial Respiration, ROS, and Antioxidant Enzymes

Curcumin attenuates oxidative stress in both animal models and cell-culture systems in which ROS generation has been induced by various stimuli, including chemical agents or pathological conditions (e.g., hyperglycemia, neurotoxicity, and aging) (Table 1). Administration of curcumin reduces oxidative damage, as evidenced by decreases in biomarkers such as malondialdehyde (MDA), a lipid-peroxidation product, and protein carbonyl content. These antioxidant effects are intimately linked to curcumin’s ability to protect against mitochondrial dysfunction elicited by these stressors, since it enhances the efficiency of mitochondrial respiration and preserves mitochondrial membrane potential. Moreover, curcumin induces both the expression and activity of SOD, CAT, GPx, and glutathione reductase (GR), thereby reinforcing the endogenous antioxidant defense system.

A representative study by Moselhy et al. [54], evaluated cellular toxicity and mitochondrial dysfunction during cellular senescence in rat brain tissue exposed to γ-radiation, and assessed the therapeutic potential of curcumin nanoparticles. The researchers found that cranial irradiation induced ROS production and oxidative stress in rat brain, as indicated by a significant increase in MDA concentrations and a significant decrease in antioxidant biomarkers, namely SOD activity, reduced glutathione (GSH) content, and total antioxidant capacity, compared with controls. Furthermore, activities of mitochondrial Complexes I and II and ATP production were diminished. In contrast, rats receiving oral curcumin (10 mg/kg) for eight weeks post-irradiation exhibited significantly lower MDA levels and significantly higher SOD activity and GSH content. These animals also demonstrated significantly increased activities of mitochondrial complexes and ATP production relative to irradiated controls; notably, Complex II activity was fully restored to control levels following curcumin treatment [54].

However, other investigations indicate that direct addition of curcumin to isolated mitochondria or to cells in the absence of a stressor may exert the opposite effect on redox balance. Curcumin appears to possess both antioxidant and pro-oxidant properties, a duality that depends primarily on its concentration and the cell type. For example, Yu et al. [64] reported that, in mouse C2C12 myoblasts under basal conditions, low curcumin concentrations (1–5 µM) did not alter cell morphology or viability but did elicit a mild increase in ROS levels associated with elevated mitochondrial mass and membrane potential. Conversely, cells incubated with higher curcumin concentrations (10, 20, and 50 µM) underwent marked morphological changes, rounding and detachment from the culture substrate, indicative of cell death. These high-dose treatments decreased viability and raised ROS levels. Specifically, exposure to 20 µM curcumin induced mitochondrial permeability transition pore opening, cytochrome c release, caspase 9 and 3 activation, and ultimately apoptotic cell death [64].

Similarly, Atsumi et al. [67] demonstrated that, in normal human gingival fibroblasts, curcumin can also act as a pro-oxidant: incubation with 10–50 µM curcumin provoked significant ROS generation, which was accompanied by loss of mitochondrial membrane potential, an early indicator of mitochondrial dysfunction, and externalization of phosphatidylserine, a hallmark of apoptosis. All induced damage was mitigated by the addition of exogenous antioxidants, such as GSH and N-acetyl-L-cysteine (NAC), suggesting that ROS mediated curcumin’s cytotoxicity [67].

Although definitive conclusions cannot be drawn from current evidence, findings from animal models suggest that high doses of curcumin (up to 400 mg/kg body weight) may exert mitoprotective effects in the presence of a stressor [62], likely due to the physiological complexity of these organisms. In contrast, in vitro studies have shown a biphasic response that depends both on curcumin concentration and the presence of a stress-inducing stimulus. When such a stimulus is present, concentrations between 10 and 20 µM appear sufficient to trigger a mitoprotective effect [65,66]. However, in the absence of a stressor, cytotoxic effects have been observed at concentrations exceeding 10 µM [64,67]. These findings highlight the need for further studies to clarify the direct effects of curcumin on cells and mitochondria under basal (non-stressed) conditions in order to better define the threshold between its cytoprotective and cytotoxic actions.

#### 5.2.2. Curcumin’s Interaction with Mitochondrial Components and Its Modulation of Membrane Fluidity

The lipophilic nature of curcumin and its poor solubility in water allow it to interact with cellular membranes. However, there is still no consensus regarding curcumin’s preferential location within the lipid bilayer, nor about its precise effects on membrane properties. Among the most relevant findings is that curcumin affects membrane fluidity; both an increase in acyl chain order across various membrane models [68,69,70] and a bilayer fluidization after curcumin administration have been reported [71].

In a recent study, the interaction of curcumin with membranes and its ability to modify membrane barrier properties, such as water permeability, was investigated using protein-free model membranes composed of 1,2-dioleoyl-sn-glycero-3-phosphocholine (DOPC). The findings revealed a concentration-dependent biphasic effect: water permeability decreased at low concentrations of curcumin (up to 2 mol%), while at higher concentrations (above 3 mol%), permeability increased. Based on these results and complementary biophysical techniques, the authors proposed that at low concentrations, curcumin interacts at the lipid–water interface through hydrogen bonding with phosphate head groups, which reduces membrane fluidity and sterically hinders the passage of water molecules, ultimately leading to decreased water permeability. In contrast, at higher concentrations, curcumin may penetrate the bilayer and interact with acyl chains, increasing membrane fluidity and, consequently, water permeability [72].

Moreover, curcumin has shown significant interactions with cardiolipin, a phospholipid found in the mitochondrial membrane of eukaryotic cells and in bacterial membranes. A study investigating curcumin’s interaction with model membrane systems, specifically fully hydrated bilayers and Langmuir monolayers composed of dimyristoylphosphatidylcholine (DMPC) or a mixture of DMPC with 4 mol% tetramyristoylcardiolipin (TMCL), used biophysical techniques such as spectrophotometry and fluorescence to assess the compound’s behavior. The authors demonstrated that curcumin associates with the polar/apolar interfaces of lipid bilayers, and that this association is enhanced by the presence of cardiolipin. Additionally, atomic force microscopy revealed that at high curcumin concentrations (10 mol%), aggregates form within DMPC monolayers, suggesting oversaturation of the system and potential disruption of membrane stability. However, in the presence of TMCL, aggregate formation was reduced, contributing to membrane stability [73].

In biomimetic models mimicking the inner mitochondrial membrane, it was observed that curcumin readily incorporates into the bilayer in the presence of cardiolipin. This behavior appears to be related to cardiolipin’s tendency to adopt a non-lamellar hexagonal phase, which may facilitate more effective curcumin association and insertion. Furthermore, curcumin insertion into the bilayers reduces lipid packing order and increases membrane fluidity. These findings are particularly relevant given that several mitochondrial membrane proteins, including cytochrome c and respiratory complexes, are closely associated with cardiolipin. Therefore, changes in membrane fluidity may affect the activity of these enzymes and, consequently, mitochondrial function. Indeed, the authors suggest that curcumin’s interactions with mitochondrial membranes, mediated by cardiolipin, could underlie its high efficacy against a broad range of metabolic diseases [71].

In addition to its effects mediated by alterations in mitochondrial membrane fluidity, curcumin has also been shown to influence mitochondrial proteins involved in the regulation of mitochondrial dynamics. For instance, in a recent study using an animal model of sepsis, curcumin was reported to inhibit the mitochondrial translocation of dynamin-related protein 1 (DRP1), a key GTPase involved in mitochondrial fission. This inhibition was associated with activation of the SIRT1 pathway, which not only reduced mitochondrial fragmentation but also promoted mitochondrial biogenesis [63].

Complementarily, in an in vitro model of heat stress using mouse C2C12 myoblasts, curcumin induced a moderate increase in non-mitochondrial ROS levels, generated via NADPH oxidase. This increase functioned as a signal that directly regulated the expression of proteins involved in mitochondrial dynamics. Specifically, an upregulation of mitofusin 2 (MFN2) and dynamin-like 120 kDa mitochondrial protein OPA1 (OPA1) was observed—proteins responsible for the fusion of the outer and inner mitochondrial membranes, respectively. Additionally, DRP1 expression was downregulated, preventing mitochondrial fragmentation. Taken together, these findings indicate that curcumin promotes mitochondrial fusion, thereby increasing the number of elongated and tubular mitochondria. This morphology has been proposed as a mechanism that enhances ATP production efficiency, potentially representing a pro-survival adaptation to thermal stress [74].

Furthermore, in a model of oxidative stress-induced damage using H_2_O_2_ in R28 retinal neuronal cells, pretreatment with 5 µM curcumin was shown to attenuate the H_2_O_2_-induced overexpression of DRP1, while simultaneously preventing the reduction in expression of the mitochondrial fusion protein MFN2. Curcumin pretreatment also preserved mitochondrial morphology and led to a reduction in intracellular ROS production. Additionally, curcumin attenuated caspase-3 cleavage and decreased cellular apoptosis. These findings suggest that curcumin may exert a neuroprotective effect against oxidative damage through modulation of mitochondrial dynamics [75]. These findings suggest that some of curcumin’s mitochondrial actions are due to both specific effects on key proteins and changes in the lipid microenvironment.

## 6. Intrinsic Apoptosis

Curcumin exhibits broad therapeutic potential, including notable antiproliferative properties. Multiple studies have demonstrated its efficacy against various cancer types, such as lung [76], kidney [77], breast [78], pancreatic [79], and colon tumors [80]. This effect is partly attributed to its preferential uptake by tumor cells over normal cells [81,82], which enhances its ability to induce cell cycle arrest, autophagy, and apoptosis [83].

Apoptosis, a tightly regulated form of programmed cell death essential for tissue homeostasis, occurs via two main pathways: the intrinsic (mitochondrial) and the extrinsic (death receptor) pathways [84]. The intrinsic pathway is triggered by internal stress signals such as elevated ROS levels and DNA damage, leading to mitochondrial outer membrane permeabilization (MOMP), cytochrome c release, apoptosome formation, caspase activation, and ultimately cell death. This pathway is critically regulated by the Bcl-2 protein family, comprising pro-apoptotic (Bax, Bad, Bak) and anti-apoptotic (Bcl-2, Bcl-xL) members that govern MOMP [85].

Treatment of various tumor cell lines with curcumin consistently alters ROS levels, decreases mitochondrial membrane potential, and upregulates pro-apoptotic markers such as Bax and cytochrome c, while downregulating anti-apoptotic proteins (see Table 2). These findings highlight mitochondrial membrane depolarization as a key event in ROS-mediated apoptotic signaling, positioning mitochondria as central players in curcumin’s anticancer action.

This mechanism is exemplified by a study conducted by Bao et al. [86], in which human ovarian cancer cell lines HO9810 and OVCAR3 were treated with 5–30 µM curcumin. Treated cells detached and floated, with the number of floating cells increasing in a concentration-dependent manner, indicating enhanced cell death. After 48 h of treatment, intracellular ROS levels rose significantly. Curcumin also elevated mitochondrial H_2_O_2_ and O_2_^−^ levels, reduced mitochondrial membrane potential, and decreased ATP production. Notably, pre-treatment with the antioxidant N-acetylcysteine (NAC) prevented both oxidative stress and mitochondrial dysfunction, suggesting that curcumin-induced apoptosis in ovarian cancer cells is largely driven by oxidative stress [86].

While many studies have focused on evaluating the pro-apoptotic effects of curcumin in various tumor cell lines, it is also important to understand its impact on normal and/or stem/progenitor cells. At low concentrations (1–20 µM), curcumin has been shown to preserve mitochondrial integrity, prevent the loss of membrane potential, and reduce cytochrome c release across different cell types. For example, in bone marrow-derived stem cells (BMSCs), curcumin combined with hypoxic preconditioning enhanced mitochondrial quality by promoting both mitochondrial fusion and complex I enzymatic activity. It also supported cell survival and inhibited mitochondrial cytochrome c release, thereby suppressing the intrinsic apoptotic pathway, as evidenced by reduced caspase-3 activation. These effects were closely associated with activation of the PGC-1α/SIRT3/HIF-1α pathway [93].

Furthermore, curcumin pretreatment was reported to protect human periodontal ligament stem cells (hPDLSCs) from oxidative stress-induced injury by enhancing cell proliferation, reducing ROS levels, and significantly decreasing apoptosis [94]. Similarly, in mouse lung mesenchymal stem cells (LMSCs) exposed to H_2_O_2_, treatment with 10 µM curcumin restored mitochondrial membrane potential, decreased ROS production, and reduced caspase-3 activation. These effects were linked to positive modulation of the AKT/Nrf2/HO-1 signaling pathway [95].

Collectively, these findings support a dual role of curcumin: on one hand, it promotes mitochondrial apoptosis in cancer cells through ROS-induced mitochondrial dysfunction; on the other, it exerts a cytoprotective effect in progenitor cells under stress conditions by maintaining mitochondrial homeostasis and attenuating apoptotic signaling. This highlights the therapeutic potential of curcumin in both oncology and regenerative medicine.

## 7. Conclusions and Future Perspectives

Several studies have demonstrated that curcumin attenuates oxidative stress in models where the production of ROS has been induced by various factors. This antioxidant effect appears to be closely associated with its ability to preserve mitochondrial function, by improving respiratory efficiency and maintaining membrane potential.

However, in the absence of a stressor, curcumin can exert pro-oxidant effects when directly applied to cells or mitochondria, leading to apoptosis. These effects depend primarily on curcumin concentration and cell type and have gained attention due to their potential application in cancer therapies, given that curcumin tends to accumulate at higher concentrations in tumor tissues.

In this context, increased ROS levels, upregulation of pro-apoptotic markers, and a reduction in mitochondrial membrane potential, an essential event in oxidative stress-mediated apoptotic signaling, have been observed.

Recently, interest has grown in understanding how curcumin interacts with mitochondria, particularly at the membrane level. Studies using lipid bilayer models suggest that curcumin insertion into the membrane is facilitated by cardiolipin, resulting in reduced lipid ordering and increased bilayer fluidity. These changes may affect the activity of mitochondrial enzymes, such as respiratory complexes, by altering the lipid microenvironment.

Together, these findings highlight the central role of mitochondria not only as targets of curcumin’s effects but also as mediators of its cytoprotective or cytotoxic actions, depending on the experimental context. However, the potential clinical application of these effects still faces significant challenges, mainly due to curcumin’s low systemic bioavailability, which limits its therapeutic efficacy. Although various strategies have been proposed to improve its pharmacokinetic profile (such as co-administration with piperine, the use of nanoparticles, and alternative routes of administration), its efficacy and safety in humans still require validation.

Therefore, it is essential to address these limitations by developing well-structured and context-specific protocols that allow for rigorous evaluation and reliable establishment of curcumin’s therapeutic potential as a modulator of mitochondrial function.

## Figures and Tables

**Table 1:** In vivo and in vitro studies on the effects of curcumin in diseases associated with mitochondrial dysfunction.

Experimental Model	Curcumin Dose/Concentration	Impact of Stress Condition on Mitochondria	Effect of Curcumin and/or Its Metabolites	Antioxidant Enzymes or Endogenous Antioxidants Involved	Ref.
**In vivo studies**					
Cellular senescence in rat brain tissue exposed to γ-radiation	Curcumin nanoparticles, 10 mg/kg in 1 mL water, three times/week for 8 weeks	↑ lipid peroxidation (MDA); ↓ antioxidant biomarkers; ↓ activities of mitochondrial complexes I and II; ↓ ATP production	↓ MDA; ↑ activities of mitochondrial complexes and ATP production	↑ SOD activity; ↑ GSH content	[54]
Myocardial infarction in rats	120 mg/kg for 4 weeks	↓ mitochondrial DNA content; ↓ activities of Complexes I and V; ↑ MDA in cardiac tissue	↑ mitochondrial DNA content; ↑ activities of Complexes I and V; ↑ ATP content	↑ SOD and GPx activities	[55]
Rotenone-induced Parkinson’s in mice	80 mg/kg for 35 days	Impaired activities of Complexes I–V; ↑ MDA in substantia nigra	↑ activities of all mitochondrial complexes; ↓ MDA in substantia nigra	↑ CAT activity via Nrf2 induction	[56]
Five-sixths nephrectomy (5/6 Nx) in rats	60 mg/kg for 7 days pre-5/6 Nx	↑ H_2_O_2_ production; ↓ activities of Complexes I and V; ↓ antioxidant enzymes; ↑ protein–MDA adducts	↓ H_2_O_2_; ↑ respiratory complex activities; ↓ protein–MDA adducts	Preservation of SOD and GPx activities	[57]
Diabetic (db/db) mice	60 mg/kg for 4 weeks	↑ mitochondrial O_2_ consumption in liver and kidney; ↑ mitochondrial MDA; ↓ mitochondrial NO synthesis in liver	In kidney mitochondria, restores state-4 and state-3 O_2_ consumption to wild-type levels; ↓ TBARS in both organs; ↑ ATPase activity and NO synthesis in liver	Not determined	[58]
Age-related mitochondrial dysfunction in rat brain	*Curcuma longa* rhizome extract (78.1% curcumin), 100 mg/kg for 3 months	↓ activities of mitochondrial complexes; ↓ total ATP content; structural damage to cristae and membranes	↑ complex activities; restores ATP content to 86% of young controls; ↓ prevalence of damaged mitochondria	Not determined	[59]
Obese mice with hepatic steatosis	Diet supplemented with 1% or 3% curcumin	↓ hepatic mitochondrial DNA expression and of biogenesis genes (e.g., NRF1, TFAM); ↓ ATP; ↑ TBARS	Normalizes expression of mitochondrial biogenesis genes; ↓ TBARS; ↑ ATP	↑ hepatic GSH expression	[60]
Aluminum-induced neurotoxicity in rats	50 mg/kg i.p.	↓ activities of Complexes I, II and IV in cortex, midbrain and whole brain; ↓ ATP synthesis; ↓ GSH in cortex and midbrain	Restores activities of all three complexes in all regions; ↑ ATP synthesis	↑ GSH content in cortex and midbrain	[61]
Potassium dichromate (K_2_Cr_2_O_7_)-induced renal oxidative damage in rats	Curcumin 400 mg/kg for 10 days pre-K_2_Cr_2_O_7_	↑ lipid peroxidation and protein carbonyls; ↓ aconitase and mitochondrial complex activities (except IV); ↓ total GSH	↓lipid peroxidation and protein carbonyls; prevents ↑ complex activities ↑ GSH	↑ CAT, GPx and SOD activities	[62]
**In vitro studies**					
LPS-stimulated HL-1 cardiomyocytes (sepsis model)	Curcumin 20 µM	LPS induced mitochondrial morphological changes; ↓ mitochondrial membrane potential; ↑ ROS	Restores mitochondrial morphology from punctate to filamentous; partially restores network structure; ↑ membrane potential; ↓ ROS	Not determined	[63]
Mouse C2C12 myoblasts	Curcumin 5 and 20 µM	—	At high [curcumin]: ↑ ROS; ↑ mitochondrial permeability; cytochrome c release; caspase 9 and 3 activation; cell death. At low [curcumin]: ↑ ROS-dependent mitochondrial mass and membrane potential	Not determined	[64]
H_2_O_2_-induced oxidative stress in rat bone marrow MSC	Curcumin 5 and 10 µM	↓ mitochondrial membrane potential; ↓ cellular ATP; ↑ intracellular ROS	↑ membrane potential; ↑ ATP; ↓ ROS accumulation	Not determined	[65]
Isolated mouse brain mitochondria treated with t-butyl hydroperoxide	Curcumin 100–500 µM	↑ lipid peroxidation; ↑ protein carbonylation; loss of membrane integrity (mPTP opening)	↓ lipid peroxidation and protein carbonylation; prevents mPTP opening	↑ endogenous GSH levels	[66]

ATP, Adenosine triphosphate; ATPase, Adenosine triphosphatase; CAT, Catalase; GSH, Glutathione; GPx, Glutathione peroxidase; HL-1, Mouse atrial cardiomyocyte cell line; i.p., intraperitoneal (injection); LPS, lipopolysaccharide; MDA, Malondialdehyde; mPTP, Mitochondrial permeability transition pore; MSC, Mesenchymal stem cells; NO, Nitric oxide; NRF1, Nuclear respiratory factor 1, O_2_, Oxygen; ROS, Reactive oxygen species, SOD, Superoxide dismutase; TBARS, Thiobarbituric acid reactive substances; TFAM, Transcription factor A, mitochondrial. ↑ indicates increase; ↓ indicates decrease

**Table 2:** Main effects of curcumin on mitochondria in different tumor cell lines.

Cell Type	Curcumin (and Derivatives) Concentration	Effect on Mitochondria	Ref.
**HO9810, OVCAR3 (ovarian cancer cells)**	5–30 µM	Decrease in mitochondrial membrane potential; increased mitochondrial O_2_^−^ and H_2_O_2_ levels. Imbalance between Bax and Bcl-2 protein levels. Elevated levels of cleaved caspase-3.	[86]
**MCF-7 (breast cancer cells)**	14 µM	Sensitizes cells to doxorubicin, enhancing ROS generation and decreasing mitochondrial membrane potential. Upregulation of Bax, Bak, and caspase-3 genes; significant downregulation of Bcl-2.	[87]
**HT-29 (human colon cancer cells)**	10 and 40 µM	Significant time- and concentration-dependent decrease in mitochondrial membrane potential; increased intracellular ROS production.	[88]
**B16-F10 (mouse melanoma cells) L-929 (mouse fibroblasts)**	2.5–50 µM	In both tumor and normal cells, a concentration-dependent increase in cleaved caspase-3 and Bax expression, with decreased Bcl-2 expression. Significant loss of mitochondrial membrane potential from 40 µM in tumor cells.	[89]
**A549 (lung cancer cells) Mitocurcumin**	2.5–10 µM	Increased Bax levels and concomitant decrease in Bcl-2 levels. Depletion of mitochondrial GSH. Loss of mitochondrial membrane potential, increased cytochrome c levels, and enhanced caspase-3 activity.	[90]
**Huh-7 (human hepatoma-derived cells)**	5–30 µM	Decreased mitochondrial membrane potential after 24 h of incubation. At 50 µM, 92% of cells exhibited low membrane potential. The decrease was associated with increased O_2_^−^ and H_2_O_2_ levels.	[91]
**HT-29 (human colon cancer cells)**	10–80 µmol/L	Decreased expression of Bcl-2 and Bcl-xL. Cytochrome c release followed by activation of caspase-3.	[92]

Bcl-2, Apoptosis regulator Bcl-2; H_2_O_2_, Hydrogen peroxide; O_2_^−^, Superoxide radical; ROS, Reactive oxygen species; SOD, Superoxide dismutase.

## Data Availability

No new data were created or analyzed in this study.

## Abbreviations

The following abbreviations are used in this manuscript:

ARE	Antioxidant response elements
ATP	Adenosine triphosphate
ATPase	Adenosine triphosphatase
Bcl-2	Apoptosis regulator Bcl-2
BMSCs	Bone marrow-derived stem cells
CAT	Catalase
DMPC	Dimyristoylphosphatidylcholine
DOPC	1,2-dioleoyl-sn-glycero-3-phosphocholine
DRP1	Dynamin-related protein 1
FADH_2_	Flavin adenine dinucleotide in its reduced form
FDA	Food and Drug Administration
GPx	Glutathione peroxidase
GR	Glutathione reductase
GSH	Glutathione
GTP	Guanosine triphosphate
H_2_O_2_	Hydrogen peroxide
hPDLSCs	Human periodontal ligament stem cells
HL-1	Mouse atrial cardiomyocyte cell line
HPLC	High-performance liquid chromatography
i.p.	Intraperitoneal (injection)
Keap1	Kelch-like ECH-associated protein 1
LPS	Lipopolysaccharide
MAMs	Mitochondrial-associated membranes
MFN2	Mitofusin 2
MDA	Malondialdehyde
MOMP	Mitochondrial outer membrane permeabilization
mPTP	Mitochondrial permeability transition pore
MSC	Mesenchymal stem cells
MSCs	Mouse lung mesenchymal stem cells
NAC	N-acetylcysteine
NADH	Nicotinamide adenine dinucleotide in its reduced form
NO	Nitric oxide
NRF1	Nuclear respiratory factor 1
Nrf2	Nuclear factor erythroid 2–related factor 2
O_2_	Oxygen
O_2_^−^	Superoxide radical
OH	Hydroxyl groups
OPA1	Dynamin-like 120 kDa mitochondrial protein OPA1
PLGA	Poly(lactic-co-glycolic acid)
ROS	Reactive oxygen species
rRNA	Ribosomal RNA
SOD	Superoxide dismutase
SULT	Sulfotransferase isoenzymes
TBARS	Thiobarbituric acid reactive substances
TCA	Tricarboxylic acid
TFAM	Transcription factor A, mitochondrial.
TMCL	Tetramyristoylcardiolipin
tRNA	Transfer RNA
UGTs	5’-diphospho-glucuronosyltransferases
